# OD23 Evolving Prostate Cancer Screening Strategies In Germany: A Cost–Utility Analysis Comparing Traditional And Emerging Modalities

**DOI:** 10.1017/S0266462324001557

**Published:** 2025-01-07

**Authors:** Muchandifunga T. Muchadeyi, Shuang Hao, Karla Hernandez-Villafuerte, Shah Khan, Nikolaus Becker, Agne Krilaviciute, Petra Seibold, Roman Gulati, Peter Albers, Michael Schlander, Mark Clements

## Abstract

**Introduction:**

In 2021, approximately 15,000 men in Germany died from prostate cancer (PCa). The national health policy is considering shifting from annual digital rectal examination (DRE)-based screening to an age-related prostate-specific antigen (PSA)-based risk-adapted and organized screening strategy. Our research investigated the cost–utility of the current DRE-based strategy versus organized age-related PSA-based risk-adaptive PCa screening strategies in Germany.

**Methods:**

We adapted the Swedish Prostata model to the German context, recalibrating it with PCa clinical and epidemiological data from the national and state registries. The model includes preclinical and clinical disease health states defined by tumor, nodal, and metastatic stages and Gleason scores, and assumes that the benefits of screening arise from stage shift. We assessed the cost–utility of 14 strategies, ranging from no screening to DRE, and age-related, PSA-based, risk-adapted screening. Health state utility values and test characteristics were sourced from the literature. Inpatient and outpatient care costs were derived from the German diagnostic-related groups and uniform-based valuation systems.

**Results:**

Among all strategies evaluated and compared with no screening, the “DRE only” strategy led to substantial overdiagnosis, the highest incremental cost, and minimal quality-adjusted life years (QALY) gains. PSA testing starting at 50 to 60 years with reflex MRI for PSA greater than 3 ng/mL cases followed by combined systemic and targeted biopsy reduced the number of biopsies and overdiagnosis by 75 percent and 26 percent, albeit for fewer QALYs and higher costs (dominated) than the same strategy without reflex MRI. The PSA-based risk-adaptive strategy, starting at 50 to 60 years without reflex MRI, demonstrated an 85 percent probability of being cost effective within the EUR30,000 (USD32,211) to EUR100,000 (USD107,369)/QALY willingness-to-pay range.

**Conclusions:**

While Germany’s HTA emphasizes clinically added benefits and health-related quality of life, cost-effectiveness analysis substantiates this evidence. As a standalone early detection tool, DRE leads to substantial overdiagnosis, unnecessary biopsies, and increased healthcare costs. Overall, this study demonstrated the importance of age-related PSA risk-adaptive PCa screening. The value of MRI deserves further investigation, considering MRI’s positive effect on screening acceptability.

